# Cytochrome c Oxidase Inhibition by ATP Decreases Mitochondrial ROS Production

**DOI:** 10.3390/cells11060992

**Published:** 2022-03-14

**Authors:** Rabia Ramzan, Amalia M. Dolga, Susanne Michels, Petra Weber, Carsten Culmsee, Ardawan J. Rastan, Sebastian Vogt

**Affiliations:** 1 Mitochondrial Bioenergetics’ Lab, Department of Heart Surgery, University Hospital of Giessen and Marburg (UKGM), Baldingerstrasse 1, D-35043 Marburg, Germany; weberpe@staff.uni-marburg.de (P.W.); a.rastan@hkz-rotenburg.de (A.J.R.); 2 Faculty of Mathematics and Natural Sciences, University of Groningen, Nijenborgh 49747 AG, 9713 Groningen, The Netherlands; a.m.dolga@rug.nl; 3 Institute of Pharmacology and Clinical Pharmacy, Philipps-University Marburg, D-35043 Marburg, Germany; susanne.michels@pharmazie.uni-marburg.de (S.M.); culmsee@uni-marburg.de (C.C.)

**Keywords:** cytochrome c oxidase, ATP, mitochondrial membrane potential, ROS

## Abstract

This study addresses the eventual consequence of cytochrome c oxidase (CytOx) inhibition by ATP at high ATP/ADP ratio in isolated rat heart mitochondria. Earlier, it has been demonstrated that the mechanism of allosteric ATP inhibition of CytOx is one of the key regulations of mitochondrial functions. It is relevant that aiming to maintain a high ATP/ADP ratio for the measurement of CytOx activity effectuating the enzymatic inhibition as well as mitochondrial respiration, optimal concentration of mitochondria is critically important. Likewise, only at this concentration, were the differences in ΔΨ_m_ and ROS concentrations measured under various conditions significant. Moreover, when CytOx activity was inhibited in the presence of ATP, mitochondrial respiration and ΔΨ_m_ both remained static, while the ROS production was markedly decreased. Consubstantial results were found when the electron transport chain was inhibited by antimycin A, letting only CytOx remain functional to support the energy production. This seems to corroborate that the decrease in mitochondrial ROS production is solely the effect of ATP binding to CytOx which results in static respiration as well as membrane potential.

## 1. Introduction

Life requires energy, which is mostly supplied by ATP. The energy demand is not a steady state process. Adaption to the needs of biological systems is vitally important as well, e.g., in case of cardiac or skeletal muscles contraction. Thus, oxidative phosphorylation has to be highly regulated in eukaryotic cells because the requirement of ATP utilization may change by a factor of 5–10 depending on the energy demand [1,2]. On the contrary, the cell also faces ultimate deleterious effects of excessive respiration in the form of an increased accompanied production of reactive oxygen species (ROS), resulting in oxidative stress. Lower numbers of ROS are suggested to incur positive effects by acting as intracellular messengers, while higher production of ROS results in aging and the development of degenerative diseases [3,4,5]. Mitochondria are the major source of ATP production on one hand and the ROS production on the other hand, the excess of which determines the cell fate, i.e., life or death [6]. The role of cytochrome c oxidase in the regulation of respiration and membrane potential via excessive ATP production has already been proposed and described [7] apart from binding of different ligands and their roles in switching between active and relaxed state of the enzyme along with molecular conformational change [8]. In case of ATP, its binding to cytochrome oxidase is of particular importance because this is the only enzyme among all respiratory chain super complexes where seven binding sites for ATP or ADP, and additionally three sites only for ADP, have been found. Therefore, its activity is highly regulated by the cytosolic as well as matrix ATP/ADP ratios [9]. It is relevant that a regulatory missing link was found in the form of a mechanism where CytOx was inhibited at high ATP/ADP ratios, and as a result ΔΨ_m_ is kept low [10,11,12]. Mitochondrial membrane potential (ΔΨ_m_) and reactive oxygen species (ROS) production are correlated in a way that ROS production at complexes I, II and III increases with an increase in mitochondrial membrane potential >140 mV [13,14]. Hence, it was proposed that this new extension of Mitchell’s theory presented in the form of second mechanism of respiratory control prevents the excessive ROS production [15].

However, in relation to ROS production, the impact of this regulated respiration and membrane potential via cytochrome oxidase in the presence of excessive ATP (ATP inhibition of the enzyme) was proposed, but has never been shown to date. Thus, in this study, we show and explain the importance of cytochrome oxidase inhibition by ATP in regulating mitochondrial ROS production.

## 2. Materials and Methods

### 2.1. Animals

All animal experiments had local approval (EX-18-2019) and were approved to compare notifiable project according to § 8a Animal Protection Act, reference V54-191020/15cMR20/16. Male Wistar rats (200–400 g) were decapitated by guillotine. Hearts were immediately excised from the chest cavities, placed into ice-cold isolation buffer (250 mM sucrose, 20 mM HEPES–neutralized with KOH to pH 7.4 at 4 °C, 1 mM EGTA, and 0.2% fatty acid-free bovine serum albumin (BSA), and washed with the same buffer to remove all the blood, until the solution became clear.

### 2.2. Isolation of Intact Rat Heart Mitochondria

Following standard isolation procedures, mitochondria were separated at 4 °C. Using a scissor, each heart was cut into small pieces in 10–15 mL of isolation buffer and homogenized by a Teflon potter in 30–40 mL of isolation buffer. The homogenate was centrifuged at 9000× *g* for 10 min. The supernatant was separated using ice-cold gauze and centrifuged at 13,000× *g* for 15 min. The obtained mitochondrial pellet was washed once by suspending the pellet in 5 mL of ice-cold isolation buffer and centrifuged again at 13,000× *g* for 10 min. Finally, the mitochondrial pellet was resuspended in 250 µL of isolation buffer and stored on ice for further use within 3–4 h. The protein concentration was estimated by the BCA (Bicinchoninic acid) method using bovine serum albumin as a standard. The stock concentrations of mitochondrial pellets were about 23.5 mg/mL ± 7.4 (*n* = 9).

### 2.3. Polarographic Measurements of Mitochondrial Respiratory Control Ratios (RCR)

Mitochondrial oxygen consumption rates were measured polarographically using a Clark-type oxygen electrode (Hansatech Oxygraph System, Norfolk, UK). The oxygen measuring chamber was calibrated at 25 °C and standard atmospheric pressure (101.32 kPa) with 1 mL of KCl-buffer (130 mM KCl, 3 mM HEPES–neutralized with KOH to pH 7.4 at 25 °C, 0.5 mM EDTA, 2 mM KH_2_PO_4_, 0.5% fatty acid-free BSA).

After calibration, 490 µL of KCl-buffer was added in the oxygen measuring chamber, followed by 10 µL of mitochondrial suspension from the stock solution. The Oxygraph chamber was closed and the oxygen measurement was started. Respiration was initiated by adding 5 mM glutamate + 5 mM malate used as substrates. The state 3/state 4 respiration rates were monitored after the addition of 0.2 mM ADP until it was dissipated. The measured respiratory control ratios were found to be 4.4 ± 0.95.

### 2.4. Polarographic Measurements of CytOx Kinetics

For kinetics measurements of CytOx activity, the oxygen-measuring chamber was calibrated at 25 °C and standard atmospheric pressure (101.32 kPa) with a kinetics measuring buffer (250 mM sucrose, 10 mM HEPES–neutralized with KOH to pH 7.2, 5 mM MgSO_4_, 0.2 mM EDTA, 5 mM KH_2_PO_4_, 0.5% fatty acid-free BSA). As indicated in the legends to the figures, required rat heart mitochondrial concentrations were added to the measuring chamber in a total volume of 0.5 mL of kinetics measuring buffer. The activity of CytOx in mitochondria was either assayed directly or in the presence of 5 mM ADP or 5 mM ATP + ATP regenerating system (RS) consisting of 160 U/mL pyruvate kinase (PK) and 10 mM phosphoenolpyruvate (PEP).

As an electron donor to cytochrome c, 18 mM ascorbate (neutralized with KOH to pH 7.0) was added to the mitochondrial sample, and measurements were started. First, the rate of oxygen uptake due to autoxidation of ascorbate was measured, followed by the CytOx activity determination in the mitochondrial sample at increasing concentrations of cytochrome c (0–40 µM). Rates of oxygen consumption were marked on the Oxygraph system to determine the CytOx activity expressed as nmol O_2_ min^−1^ mL^−1^ at the substrate concentration range of 0–40 µM cytochrome c. Later, the rate of oxygen uptake was corrected by subtracting the autoxidation rate of ascorbate (measured at the start) from all other enzymatic activity values using Microsoft Excel. Finally, the presented CytOx activity was calculated by dividing rates (nmol O_2_ min^−1^ mL^−1^ oxygen consumption) by the corresponding mg/mL mitochondrial protein concentrations. Graphs containing kinetics values of CytOx activity vs. cytochrome c concentrations were prepared using GraphPad Prism software (GraphPad Software, Inc. La Jolla, CA, USA).

### 2.5. Measurements of Mitochondrial Membrane Potential and ROS Production by Spectrofluorometry

Variations in the mitochondrial membrane potential and ROS production were measured in 96-well plates with a FLUOstar Optima fluorescence plate reader (BMG LABTECH GmbH, Germany) using DiOC_6_(3) and MitoSOX (Thermo Fisher Scientific, Darmstadt, Germany) fluorescent dyes, respectively. Following the results of CytOx kinetics at variable mitochondrial protein concentrations, two different (lowest/highest) protein concentrations of isolated intact rat heart mitochondria were taken from the stock protein solution. The lowest rat heart mitochondrial protein concentration used was about 0.054 mg/mL ± 0.004, while the highest was about 1.128 mg/mL ± 0.07. Each sample was prepared in a total volume of 0.5 mL kinetics measuring buffer. For membrane potential, 20 nM DiOC_6_(3) and for ROS measurements 2.5 µM MitoSOX fluorescent dyes, were added to each corresponding sample prior to pipetting into the 96-well plate. Further substrate additions to the mitochondrial samples are indicated in the respective figure legends. Measurements were performed in duplicates.

### 2.6. Measurements of Mitochondrial Membrane Potential and ROS Production by Flow Cytometry

In case of flow cytometry, due to the known fluorescence quenching effect [16,17], the data collected by spectrofluorometry could be verified only at low mitochondrial protein concentrations. The decrease in mitochondrial membrane potential and increase in ROS production were measured using TMRE (Thermo Fisher Scientific, Darmstadt, Germany) and MitoSox™ fluorescent dyes, respectively. The fluorescence of each dye in each individual sample was analyzed using a FACS-Guava easyCyte™ HT System (easyCyte 6–2 L, Merck Millipore, Darmstadt, Germany).

Each sample was prepared by adding 0.054 mg/mL ± 0.004 of isolated intact rat heart mitochondria in a total volume of 0.5 mL kinetics measuring buffer. Except the negative controls (samples without dyes), all other samples were incubated for 15 min at room temperature only in the presence of respective dye (0.2 µM TMRE or 2.5 µM MitoSox™). Following incubation, substrates were added as indicated in the figure legends.

### 2.7. Statistical Analysis

Statistical analysis were performed by ANOVA; either a standard one-way ANOVA with Tukey’s Post hoc analysis or a regular two-way ANOVA with Bonferroni Post-hoc test using GraphPad Prism software (GraphPad Software, Inc. La Jolla, CA, USA), and graphs were plotted. The results are presented as mean ± SD.

## 3. Results

### 3.1. ATP Effect on Mitochondrial Respiration, ΔΨ_m_ and ROS Production

**Figure 1:**
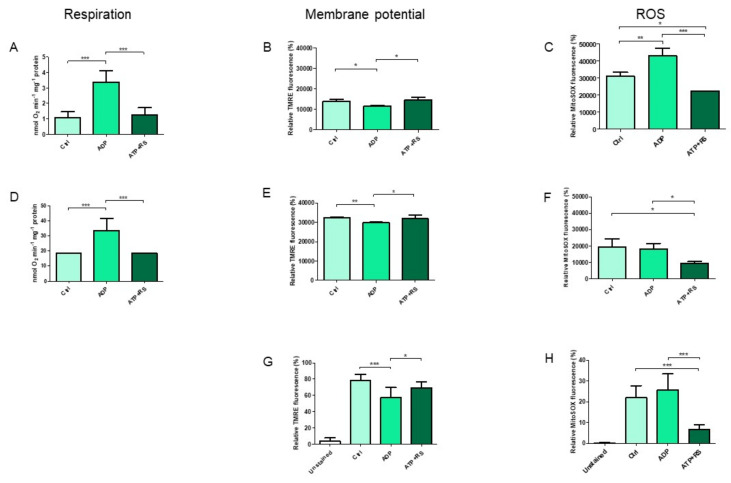
Correlation of mitochondrial respiration, membrane potential and ROS production. Mitochondrial oxygen consumption (respiration) rates were measured by polarography in the presence of endogenous fuel substrates using two different concentrations of mitochondria, i.e., 1.128 mg/mL ± 0.07 and 0.054 mg/mL ± 0.004 ((**A**,**D**), respectively) and were correlated to the corresponding spectrofluorometric measurements of membrane potential ((**B**,**E**), respectively) as well as to the ROS productions (**C**,**F**). The required mitochondrial concentration (*n* = 4 individual experiments) was added into the total volume of 0.5 mL of kinetics measuring buffer with or without substrate additions as indicated. Finally, samples were measured in duplicates after mixing with the corresponding fluorescent dyes. In addition, mitochondrial membrane potential (**G**) and ROS measurements (**H**) were also performed by flow cytometry using 0.054 mg/mL ± 0.004 (*n* = 5 individual experiments), and were compared to the corresponding polarographic measurements of mitochondrial respiration (**D**). A standard one-way ANOVA with Tukey’s Post hoc analysis was applied to the data. * *p* ≤ 0.05; ** *p* ≤ 0.01; *** *p* ≤ 0.001. The concentrations of added substrates were as follows; 5 mM ADP, 5 mM ATP + RS (10 mM phosphoenolpyruvate + 160 U/mL pyruvate kinase). Rat heart mitochondria (RHM) without addition of substrates was used as a control (Ctrl).

The addition of ADP (Figure 1A) stimulated the state 3 respiration because of the consumption of ΔΨ_m_ by ATP synthase that used it to produce ATP from added ADP. This resultant decrease in ΔΨ_m_ (Figure 1B) stimulated the respiration with the consequent significant increase in ROS production (Figure 1C) in comparison to the control. However, in the presence of ATP + RS, although respiration (state 4) and membrane potential both remained the same as that of control, ROS production in comparison to both the control and ADP was significantly decreased. In order to exclude the fact if it is due to the ATP/ADP ratio dependent on the mitochondrial protein concentration, a 20 times lower concentration of mitochondria was used to measure the respiration (Figure 1D), ΔΨ_m_ (Figure 1E) and ROS production (Figure 1F). Similar results were found in the cases of all bioenergetics parameters, except that the differences between ROS concentrations, measured in the presence of ADP and control, were non-significant at this mitochondrial concentration. The general comparison of ΔΨ_m_ measurements between two different mitochondrial concentrations (Figure 1B,E) under all conditions indicate that with 1.128 mg/mL mitochondria, the measured relative dye fluorescence (%) is less than 0.054 mg/mL mitochondria while that of the ROS measurements (Figure 1C,F) is higher at 1.128 mg/mL than 0.054 mg/mL mitochondria.

Furthermore, identical results were found in case of ΔΨ_m_ (Figure 1G) as well as ROS production (Figure 1H) in comparison to Figure 1E,F, respectively. These data show that independent of the mitochondrial concentration used for measurement, the addition of ADP stimulates the state 3 respiration, decreases the membrane potential and increases the ROS production as already known. However, the addition of ATP sustains to the control, both the respiration and membrane potential, and decreases the ROS production to the lowest.

### 3.2. Variation in CytOx Kinetics Depending on Mitochondrial Protein Concentrations

**Figure 2:**
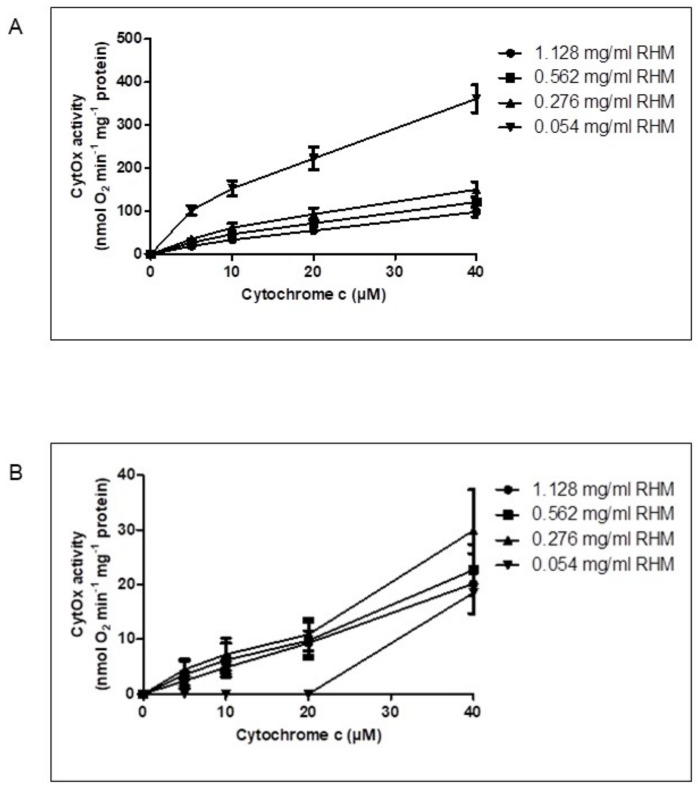
Variation in CytOx kinetics depending on mitochondrial protein concentrations. The activity of CytOx kinetics was measured using 1.128, 0.562, 0.276 and 0.054 mg/mL mitochondrial concentrations at 0, 5, 10 and 40 µM cytochrome c (substrate of CytOx) in the presence of 18 mM ascorbate, which was used to reduce cytochrome c. (**A**) in the presence of 5 mM ADP, (**B**) in the presence of 5 mM ATP + RS (10 mM PEP + 160 U/mL PK). The activity of CytOx measured in the presence of ADP as well as ATP + RS at lower (0.054 mg/mL) concentration of RHM was significantly different (*p* ≤ 0.0001) from all other higher mitochondrial protein concentrations when a regular two-way ANOVA with Bonferroni Post hoc test was applied to the data consisting of *n* = 4 individual experiments.

Subsequent to respiration measurements, the kinetics of CytOx activity in isolated intact rat heart mitochondria was measured at variable protein concentrations, i.e., 0.054, 0.276, 0.562 and 1.128 mg/mL in the presence of ADP (Figure 2A) and ATP + RS (Figure 2B) at 5, 10, 20 and 40 µM cytochrome c.

It was found that the activity of CytOx measured in the presence of ADP was almost four times higher at lower concentrations of mitochondria, i.e., 0.054 mg/mL ± 0.004, than the higher mitochondrial protein concentrations of 1.128 mg/mL ± 0.07 (Figure 2A). However, the kinetics of CytOx activity measured in the presence of ATP + RS (Figure 2B) showed that the enzyme was inhibited until 20 µM cytochrome c at lower concentrations of rat heart mitochondria (0.054 mg/mL) while at all other following mitochondrial protein concentrations (0.276, 0.562 and 1.128 mg/mL), this inhibition was already released even at the initial cytochrome c concentrations. Since ATP can be hydrolyzed to ADP as soon as it is added, this can decrease the ATP/ADP ratio in the mitochondria and thus may result in the consequent release of CytOx inhibition. Therefore, an ATP regenerating system (RS) consisting of phophoenolpyruvate and pyruvate kinase was added to keep the ATP/ADP ratio high enough so that the sufficient amount of ATP is available which can bind to CytOx and keeps the enzyme inhibited.

A regular two-way ANOVA with Bonferroni Post hoc test showed that the effect of lower concentrations of rat heart mitochondrial protein was statistically significant (*p* ≤ 0.0001) to all other increasing mitochondrial concentrations measured in the presence of either ADP or ATP + RS. Furthermore, when CytOx activity was measured in the presence of ADP, statistically significant differences were also found between 0.276 and 1.128 mg/mL mitochondria, while the differences between 0.276 and 0.562 mg/mL as well as 0.562 and 1.128 mg/mL mitochondrial concentrations were statistically non-significant (Figure 2A). The activity of CytOx measured in the presence of ATP + RS at lower mitochondrial concentration (0.054 mg/mL) was significantly different (*p* ≤ 0.0001) from all other mitochondrial concentrations. There were no statistically significant differences found among other groups of mitochondria (Figure 2B). Thus, using the optimal concentration of mitochondria (0.054 mg/mL ± 0.004) is essential for maintaining the high ATP/ADP ratio because the extent of CytOx inhibition by ATP varies with different mitochondrial concentrations. Finally, the measurement of allosteric ATP inhibition of CytOx in isolated intact rat heart mitochondria is only possible when higher ATP/ADP ratios are maintained by using the optimal mitochondrial concentration.

### 3.3. Correlation of CytOx Kinetics to ΔΨ_m_ and ROS Measurements

**Figure 3:**
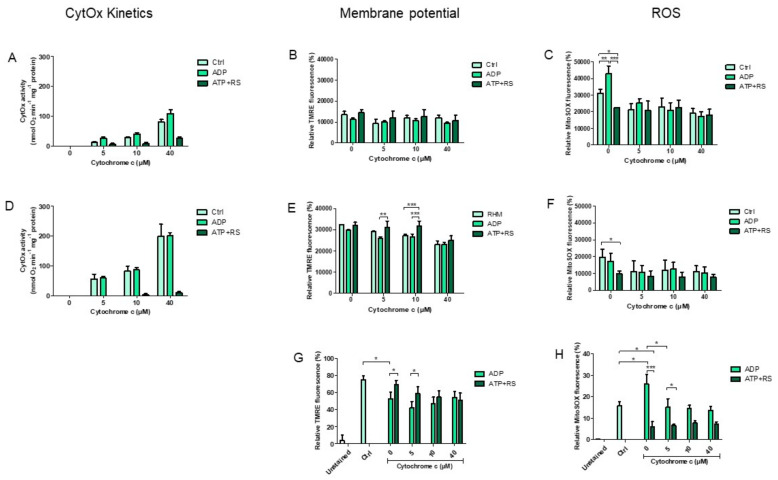
Comparison of CytOx kinetics to mitochondrial membrane potential and ROS production measurements. The kinetics of CytOx activity was measured in 1.128 mg/mL ± 0.07 mitochondria (**A**) and was correlated with the membrane potential (**B**) and ROS productions (**C**) performed by spectrofluorometry (*n* = 4 individual experiments). Similarly, measurements were also performed using 0.054 mg/mL ± 0.004 mitochondria, i.e., CytOx kinetics (**D**), ΔΨ_m_ (**E**) and ROS (**F**). Additionally, membrane potential (**G**) and ROS production (**H**) were also measured by flow cytometry (*n* = 3 individual experiments) in 0.054 mg/mL ± 0.004 mitochondria. The concentrations of added substrates were: 5 mM ADP or 5 mM ATP + RS (10 mM PEP + 160 U/mL PK). Each sample was measured at 0, 5, 10 or 40 µM cytochrome c used as a substrate for CytOx. All measurements were performed in the presence of 18 mM ascorbate which was added to reduce cytochrome c. A regular two-way ANOVA with Bonferroni Post hoc test was applied and following *p* values were found showing significant differences between different groups based on cytochrome c concentrations; *p* ≤ 0.0001 for (**A**); *p* ≤ 0.0001 for (**B**); *p* ≤ 0.0001 for (**C**); *p* ≤ 0.01 for (**D**); *p* ≤ 0.05 for (**E**) and *p* ≤ 0.0001 for (**F**). * *p* ≤ 0.05; ** *p* ≤ 0.01; *** *p* ≤ 0.001.

From the enzyme kinetics measurements, two extreme mitochondrial concentrations were selected, i.e., 1.128 mg/mL (maximum) and 0.054 mg/mL (minimum) mitochondria (Figure 3A,D, respectively). These concentrations were taken due to the greatest variation appearing in the inhibition of CytOx activity by ATP particularly from full inhibition at minimum mitochondrial concentration until the release of inhibition at the maximum mitochondrial concentration. Subsequently, these kinetics measurements were correlated with the membrane potential (Figure 3B,E) and ROS productions (Figure 3C,F). In the kinetics experiments, the control was RHM without any nucleotide additions. The CytOx kinetics measured with 1.128 mg/mL mitochondria showed that the differences between control and ADP, control and ATP, and ADP and ATP +RS (Figure 3A) were highly significant (*p* ≤ 0.0001) according to a regular two-way ANOVA with Bonferroni Post hoc analysis. Furthermore, it was found that when the enzyme kinetics was measured with 0.054 mg/mL mitochondria, the differences between ATP and RS in comparison to the control and to ADP, were also statistically significant (*p* ≤ 0.0001), while the differences between control and ADP were not significant (Figure 3D). These results indicate the particular effect of ADP and ATP nucleotides on mitochondrial oxygen consumption via CytOx where ADP stimulates the respiration, while the presence of high ATP concentrations inhibit the respiration. Moreover, this inhibitory effect of ATP is even more intense, i.e., completely inhibiting the enzyme, when the used mitochondrial sample concentration is less. This may indicate that the difference in oxygen consumption rates, based on mitochondrial concentrations used for measurements, may be due to the presence of variable ATP/ADP ratio in isolated mitochondria. The effects of various cytochrome c concentrations under all conditions in Figure 3A,D were also found to be highly significant (*p* ≤ 0.0001), indicating that the addition of external cytochrome c (used as a substrate) does reach the intermembrane space of mitochondria and influence the activity of its enzyme, i.e., CytOx.

These variabilities in kinetics of CytOx activity based on different mitochondrial protein concentrations as well as under the use of ATP and ADP, were correlated to the changes in mitochondrial membrane potential measurements (Figure 3B,E) as well as to the ROS production (Figure 3C,F); both were performed by spectrofluorometer. Although the kinetics measured at higher protein concertation (1.128 mg/mL) show that there are statistically significant differences in respiration under various conditions (Figure 3A) but at the same mitochondrial concentration, the membrane potential differences between control, ADP and ATP + RS at various cytochrome c concentrations were found to be statistically non- significant (Figure 3B). This may indicate that the mitochondrial respiration under kinetics conditions was uncoupled, i.e., oxygen consumption proceeded without energy production, so the membrane potential was sustained (neither produced further nor consumed anymore). Another explanation could be that this concentration of mitochondria is not optimal for measuring statistically significant differences in ΔΨ_m_ based on the required ATP/ADP ratio in the isolated mitochondria.

Similarly, ΔΨ_m_ measurements at a lower concentration of mitochondria, i.e., 0.054 mg/mL (Figure 3E) indicate that without any addition of cytochrome c (at 0), the differences between control, ADP and ATP + RS were not significant, although ADP or ATP + RS were present in corresponding samples. However, when cytochrome c was added at 5 and 10 µM concentrations, the differences between ADP and ATP + RS appeared to be significant (*p* ≤ 0.0001). Additionally, at 40µM cytochrome c, although differences were found to be significant (*p* ≤ 0.0001) as compared to 0, 5 and 10 µM cytochrome c, there was no significance between RHM, ADP and ATP + RS. Comparable results were found when ΔΨ_m_ was measured by FACS analyzer using 0.054 mg/mL RHM (Figure 3G).

These results demonstrate that variations in ΔΨ_m_ measurements after the addition of cytochrome c as well as ADP and ATP, were possible to measure only when the optimal mitochondrial concentration (0.054 mg/mL) was used. Moreover, at 5 and 10 µM concentration, the added cytochrome c restores/increases the ΔΨ_m_ in comparison to corresponding RHM and in the presence of ADP measurements.

In addition to kinetics and ΔΨ_m_, ROS productions were also measured in parallel at both sample concentrations of mitochondria, i.e., at higher 1.128 mg/mL and lower 0.054 mg/mL (Figure 3C,F, respectively). It is clearly evident that the addition of only ATP + RS in the absence of extra-mitochondrial cytochrome c resulted in statistically significant differences (*p* ≤ 0.01), in comparison to corresponding ADP and RHM sample measurements.

Furthermore, the addition of 5, 10 and 40 µM cytochrome c to corresponding samples of RHM as well as ADP affected the ROS production significantly (in Figure 3C, *p* ≤ 0.001 and in Figure 3F, *p* ≤ 0.01) which otherwise was higher in the absence of cytochrome c addition. The ROS concentrations remained the same in the absence and presence of cytochrome c when ATP + RS was added. These results indicate that ROS production decreases tremendously just by adding ATP + RS independent of mitochondrial concentrations as well as the presence/absence of cytochrome c. The increased ROS concentrations, in the control and in the presence of ADP, were significantly reduced to the level of those of ATP + RS just by adding cytochrome c. Corresponding results were found when ROS concentrations were measured by FACS analyzer using 0.054 mg/mL RHM (Figure 3H). Significant differences were found with and without cytochrome c as well as in the presence of ADP or ATP + RS (** *p* ≤ 0.01).

### 3.4. ATP Effect on ΔΨ_m_ and ROS Production Is Specific to CytOx

**Figure 4:**
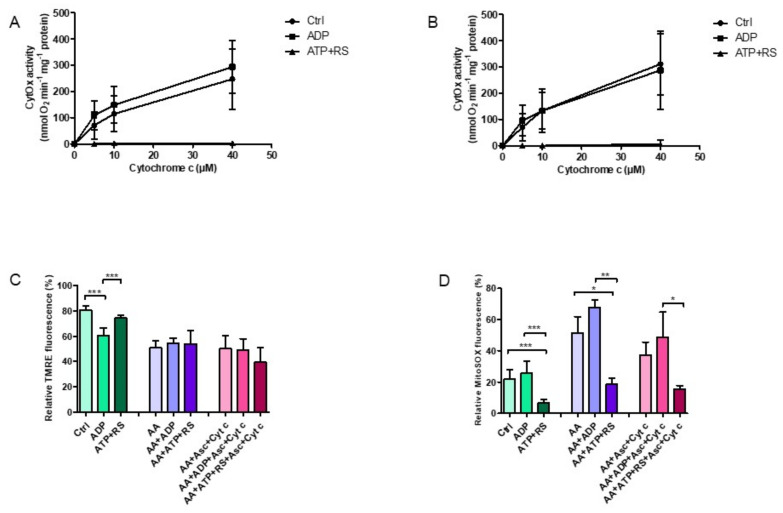
Effect of antimycin A on CytOx kinetics, mitochondrial membrane potential and ROS production. Samples were prepared by adding 0.054 mg/mL ± 0.004 freshly isolated rat heart mitochondria to a total volume of 0.5 mL kinetics measuring buffer in the presence or absence of 45 µM antimycin A. (**A**) The kinetics of CytOx activity was measured polarographically in the absence of nucleotides (control) or in the presence of 5 mM ADP or 5 mM ATP + RS (10 mM PEP + 160 U/mL PK) at increasing cytochrome c concentrations of 0, 5, 10 and 40 µM using 18 mM ascorbate to reduce cytochrome c. (**B**) The CytOx kinetics was measured in the presence of 45 µM antimycin A. For n = 5 individual experiments, a regular two-way ANOVA with Bonferroni Post hoc test was applied. Significant differences (*p* ≤ 0.0001) were found (in case of both A and B) for ATP + RS in comparison to control and ADP, while the differences between the control and ADP were not significant. Furthermore, cytochrome c concentrations were found to be significantly (*p* ≤ 0.0001) affecting the oxygen consumption rates. Mitochondrial membrane potential (**C**) and ROS production (**D**) were performed by flow cytometry. A standard one-way ANOVA with Tukey’s Post hoc analysis was performed with data from *n* = 5 measurements (* *p* ≤ 0.05; ** *p* ≤ 0.01; *** *p* ≤ 0.001).

In order to substantiate that these measured effects on ΔΨ_m_ and ROS production in isolated rat heart mitochondria were particularly related to CytOx and not to any other respiratory chain enzyme, antimycin A was used to block the mitochondrial respiration at complex III of the electron transport chain followed by measurements of CytOx kinetics (Figure 4A,B), ΔΨ_m_ (Figure 4C) and ROS productions (Figure 4D). The lower 0.054 mg/mL mitochondrial protein was used, since, at this concentration, significant changes were obtained with respect to different mitochondrial bioenergetics parameters. It was found that that the prevalence of CytOx inhibition in the kinetics, when ATP + RS were added (Figure 4A), did not change with the presence of antimycin A (Figure 4B). However, antimycin A did decrease the ΔΨ_m_ measured by FACS analyzer in all samples where added (Figure 4C) in comparison to the control group (control, ADP and ATP + RS). The addition of 2 µM cytochrome c (reduced by 18 mM ascorbate) to stimulate CytOx in the presence of antimycin A, did not show any significant differences and ΔΨ_m_ did not change.

Subsequent ROS measurements, also performed by FACS analyzer (Figure 4D), showed an increased ROS production in the presence of ADP, corresponding to the results shown already. However, these ROS concentrations were further increased when antimycin A was added. Nonetheless, the ROS levels in the presence of ATP + RS remained to the lowest, while those in the presence of ADP were the highest among all groups, independent of the presence or absence of antimycin A.

Succinctly, it is evident that even when the respiratory chain is blocked at complex III, ATP inhibition of CytOx in the enzyme kinetics remained unaffected, showing that the binding of ATP and its effect on oxygen consumption was specifically related to CytOx. Furthermore, the membrane potential under the ATP inhibitory affect was sustained, but the ROS production was tremendously decreased, similar to the results described before.

## 4. Discussion

The new extension of Mitchell’s theory proposed earlier [7], suggested that respiration in living eukaryotic organisms is usually controlled by a ΔΨ_m_-independent mechanism which is based on the inhibition of CytOx by ATP at high ATP/ADP ratios.

It is well-known that electrons which are transferred from NADH and FADH_2_, produced during tricarboxylic acid cycle, are then transported through the ETC, which drives proton pumps (complex I, II and IV, respectively) of inner membrane. The generated proton motive force consists of ΔΨ_m_ and ΔpH. In mitochondria, the electrical gradient contributes to a larger extent than the pH gradient. Finally, the electrical gradient is consumed by ATP-synthase (complex V) for synthesizing ATP from ADP and P_i_. This working circuit appears to be logically closed. However, ongoing activities such as muscle contraction, consume ATP and hydrolyze it back to ADP and P_i_. Subsequently, the ATP pool is lessened and “new ATP” is needed. Further synthesis of ATP decreases ΔΨ_m_ and the latter is regenerated by the mitochondrial proton pumps. This is the basis of Mitchell’s theory [18,19]. However, some questions remained unanswered. For example, during a heavy workload, especially in case of cardiac contractility which is a continuous and time-dependent process, a sudden rise in contractility requires an immediate high consumption of ATP and subsequent re-synthesis. Therefore, it appears obvious that an efficient regulation of the system is absolutely necessary. The functional triangle, consisting of CytOx activity, ΔΨ_m_ and the subsequent regulated ROS production, represent the platform which makes this kind of regulation possible. Under physiological conditions, very high ATP/ADP ratios are present in the cell. As known, ATP acts as an allogenic modulator [20] and thus binds extramitochondrially to the cytosolic domain of CytOx subunit IV [9] and/or intramitochondrially [21]. Additionally, cAMP-dependent protein kinases are suggested to be involved in the allosteric inhibition [22]. CytOx contains seven high-affinity binding sites for ATP or ADP and three additional sites only for ADP per monomer of the isolated enzyme [9]. The ATP binding site for any other complex of the mitochondrial respiratory chain has not been reported so far. Furthermore, at 100% intraliposomal ATP, the H^+^/e^−^ stoichiometry of a reconstituted enzyme is decreased to half when measured below 98% intraliposomal ATP (more than 2% ADP), which may have physiological consequences in maintaining the body temperature at rest or during sleep, i.e., at low ATP expenditure [23]. Thus, the observed effect of high ATP on CytOx would be expected, which may lead to partial uncoupling of mitochondrial energy transduction and in the stimulation of thermogenesis [24]. Among the three proton pumps of the respiratory chain of mitochondria and bacteria, only CytOx is known to exhibit a slip of proton pumping. Intrinsic uncoupling was already shown to increase thermogenesis at high membrane potential and at high ATP/ADP ratios [25]. As a result, conformation of the enzyme changes with ATP and activity switches into a relaxed state (lower ATP synthesis, low ROS release), although the ΔΨ_m_ remains higher while not excessively spent.

**Figure 5:**
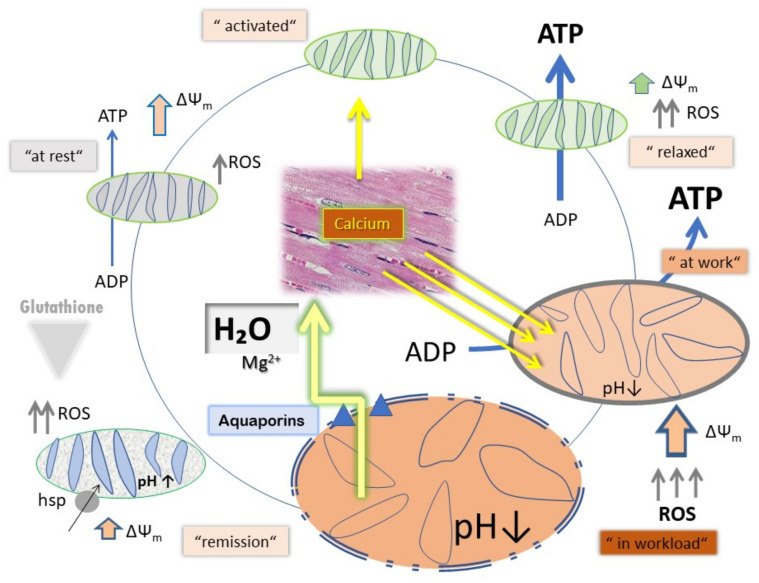
Schematic representation of variations in mitochondrial functional components under different physiological states, e.g., relaxed, activated and activated under work load.

To clarify this mechanism, in the presented experimental settings, two different concentrations of mitochondria were used in the presence of 5 mM ATP and a backup support for ATP with an ATP-regenerating system consisting of PEP and PK. With a lower mitochondrial concentration, we intended to achieve the conditions of approximately 100% ATP presence, close to the existing biological conditions in tissues. On the contrary, an increased ATP dissipation at high mitochondrial concentrations mimics biological conditions, as in case of a high workload. In ^31^P-NMR magnetization transfer measurements myocardial ATP contents were measured up to 10 mM ATP. Herein, the normal ATP synthesis rate was determined to be 29.8 ± 1.6 μmol/min/g w.w. [26]. In addition to the question of H^+^/e^−^ stoichiometry under these conditions, the experiments addressed the question of the relationship between CytOx activity, ΔΨ_m_ and ROS production, describing physiological cycling conditions. In the beating heart, ΔΨ_m_ values of 100–140 mV under physiological conditions have been explained by ATP inhibition of CytOx through feedback inhibition by ATP at high ATP/ADP ratios [12]. In particular, in the permanent working heart muscle, we have to face extreme conditions for high oxygen consumption (almost the highest, 70% oxygen extraction from the blood [27]), highest CytOx activity among all the organs (around 156 ± 9.6 nmol O_2_ min^−1^ mg protein^−1^ [28]), but almost equally high ATP contents in comparison of Kidney to Heart to Liver (ratio 1.0:0.96:0.96). The answer for this phenomenon comes from the high content of Creatine phosphate feeding the ATP pool [29] as an additional back-up system. This is not the case in our experimental system at high concentrations of mitochondria. Even with ATP and RS, the ATP/ADP ratio is not kept high enough for the ATP-dependent inhibitory effect on CytOx at relaxed state respiration. Here, although a high number of mitochondria reduce the efficiency of ATP-induced inhibition, the increased amounts of ADP cause a high production as well as consumption of ATP by the mitochondria (such as during workload), it appears doubtful if this circumstance does mean automatically “mitochondria in stress” (Figure 5). However, shifting towards the pathological status appears pending when workload is excessively progressing or limitations occur because of reduced oxygen and further substrate supply. Heat stress seems to be efficient in creating a scenario mimicking excessive oxygen demand on behalf of higher ETC activities [30,31].

Active state respiration (under stress) is switched on by signaling through ROS, hormones, peptides and/or phosphatases when excessive oxygen utilization is demanded and H^+^/e^−^—stoichiometry is approximately close to the relation of 1. Although ΔΨ_m_ increases to a lesser extent, ROS are excessively produced. At reperfusion of ischemic tissue, bursts of ROS production and calcium overload lead to apoptosis and/or necrosis [32]. This has been explained earlier as similar to the modulatory action of near-infrared light upon CytOx activity and ischemia. In a model of neuroprotection, the activity of CytOx is down-regulated under normal controlled conditions via phosphorylation while during ischemia, CytOx is dephosphorylated. The enzyme cannot operate due to the lack of O_2_, while NADH and succinate accumulate. At the onset of reperfusion and in the presence of O_2_, the ETC operates at maximal activity, creating pathologically high ΔΨ_m_ (active state), which leads to reverse electron flux, excessive ROS production at ETC complexes I and III, and mitochondrial loss of cytochrome c [33]. That is why inhibition of ETC in case of myocardial ischemia protects the tissue during reperfusion recovery [34].

ATP is the energy currency of the organism, maintaining physiological functions and survival, while ROS (excluding signaling properties) are degenerating agents, causing aging and degenerative diseases. Mitochondria lack sufficient ATP content when isolated because ADP induces respiration in both coupled and uncoupled mitochondria [35]. Thus, to measure reliable respiratory control ratios, optimal mitochondrial sample concentrations are used where high rates of oxygen consumption can be achieved in the presence of ADP. Usually sample concentrations are normalized to values higher than 1–2 mg/mL of mitochondrial protein. Moreover, depending on the quantity and the quality of the mitochondria after isolation, some research groups use much higher sample values, which in turn result in variable results with respect to membrane potential and ROS measurements. Furthermore, mitochondria localized to the surface in a solution consist of mitochondrial population with loosely coupled oxidative phosphorylation, thus show respiratory control ratios of 1.7 to 1.1. In comparison mitochondria localized deep in the solution reveal a tight-coupling state [36]. In our study, we found that to compare the membrane potential with ATP inhibition of CytOx, mitochondrial protein concentrations of 50–100 µg/mL are required because using high sample concentrations excludes the possibility of measuring the ATP inhibition of CytOx due to the decreased ATP/ADP ratio. Thus, ATP inhibition of CytOx, the mitochondrial membrane potential and ROS production can be measured and compared, even at low sample concentrations. The variability in the extent of ATP inhibition of CytOx kinetics correlates with the variable inhibition of uncoupled respiration in intact isolated mitochondria by ATP [37]. It is to be noted that ROS measurements always remained the lowest with ATP + RS at all mitochondrial concentrations, despite sustained ΔΨ_m_.

The ATP-mediated CytOx inhibition is different from CytOx inhibition by cyanide because the latter one irreversibly blocks the enzyme, thereby completely inhibiting mitochondrial respiration [38], resulting in a decrease in ATP content and a consequent increase in ROS production. Moreover, the inhibition of other complexes, such as I and III, decreases ΔΨ_m_ and increases ROS production [39], e.g., after inhibition of complex III by antimycin A [40]. We found similar results in our study in the presence of antimycin A, which decreased the mitochondrial membrane potential with the consequent increase in ROS production. However, the inhibition of electron transport chain at Complex III by antimycin A demonstrates that these high ATP-/ADP-dependent effects are solely related to CytOx and may result in a conformational change in the enzyme.

ΔΨ_m_ is an important regulator of mitochondrial ROS. The generation of ROS becomes exponentially independent of ΔΨ_m_ above 140 mV. Furthermore, ROS can stimulate mitochondrial uncoupling [41]. In our study, we show that similar results were found in the presence of endogenous substrates, or even when substrates for CytOx were added with an open or blocked ETC at complex III by antimycin A. The differences in ΔΨ_m_ and ROS production at high and low mitochondrial protein concentrations can be explained by the following factors: (i) differences in the rate of coupling and proton leakage [42] (ii) formation of supercomplexes upon mitochondrial contraction and structural compression where supercomplexes ultimately increase the pressure on the electron transport chain for more output, leading to an increased RCR. In contrast, when the mitochondria relax, their structures are more expanded or swollen; the complexes in the electron transport chain remain in individual structural forms with less supercomplex formation, resulting in low RCR values (iii) the prevalence of structural differences, because in concentrated mitochondria due to lower surface-to-volume ratio, the binding sites of individual complexes may be hidden and thus regulation can be maintained only through mitochondrial signals such as phosphorylation/dephosphorylation. While in swollen mitochondria (less mitochondrial protein in more buffer solution), an increase in surface-to-volume ratio may result in less supercomplex formation, so the binding sites of individual complexes are exposed, and their regulation is maintained by cytosolic signals e.g. as under the influence of thyroid hormones where mitochondria possess an increased respiratory activity with three times greater proton permeability of the inner membrane compared to mitochondria with low respiratory activity. Such increases in mitochondrial RCR by thyroid hormones also had an increase in the area of the inner membrane/mass protein by a factor of 2–3 [43]. Furthermore, (iv) differences in the mitochondrial concentration may relate to differences in the aerobic capacity, i.e., increasing the concentration of mitochondria at a constant energy demand may result in an increased ROS production. Finally, (v) when ETC is inhibited by ATP at CytOx, there is no blackflow of electrons, so it might be that no reverse electron transport takes place.

## 5. Conclusions

Concomitant binding of ATP or ADP to CytOx and to the ATP synthase represents a physiological signal for the regulation of mitochondrial respiration and oxidative phosphorylation. Addition of ADP into intact isolated mitochondria couples mitochondrial respiration to oxidative phosphorylation to produce more ATP, thus it stimulates mitochondrial respiration with the resultant decrease in membrane potential. On the contrary, ATP binding to complexes IV at high ATP/ADP ratio results in ΔΨ_m_-independent inhibition of enzyme activity. In our experiments, we found that ATP binding to CytOx sustains both the respiration and membrane potential in comparison to the control. This is because ΔΨ_m_ is not required to be consumed for ATP production due to the presence of sufficient ATP. So, the respiration is inhibited without an increase in ROS production. Furthermore, the suggested mitochondrial swelling and shrinking caused by variable water content affect the folding of cristae. The unfolding of mitochondrial cristae in the case of swelling results in the conversion of dimeric CytOx to monomeric form, resulting in hyperbolic enzyme kinetics. The return of mitochondrial shape to the contracted state induces back the formation of CytOx dimers, thus maintaining mitochondrial respiration in the physiological range.
